# *Lactobacillus casei* Zhang and vitamin K2 prevent intestinal tumorigenesis in mice via adiponectin-elevated different signaling pathways

**DOI:** 10.18632/oncotarget.15791

**Published:** 2017-03-01

**Authors:** Yong Zhang, Chen Ma, Jie Zhao, Haiyan Xu, Qiangchuan Hou, Heping Zhang

**Affiliations:** ^1^ Key Laboratory of Dairy Biotechnology and Engineering, Education Ministry of P. R. China, Department of Food Science and Engineering, Inner Mongolia Agricultural University, Hohhot 010018, P. R. China

**Keywords:** intestinal tumorigenesis, adiponectin, Lactobacillus casei, vitamin K2

## Abstract

The incidence of colon cancer has increased considerably and the intestinal microbiota participate in the development of colon cancer. We showed that the *L. casei* Zhang or vitamin K2 (Menaquinone-7) intervention significantly alleviated intestinal tumor burden in mice. This was associated with increased serum adiponectin levels in both treatments. But osteocalcin level was only increased by *L. casei* Zhang. Furthermore, the anti-carcinogenic actions of L. casei Zhang were mediated by hepatic Chloride channel-3(CLCN3)/Nuclear Factor Kappa B(NF-κB) and intestinal Claudin15/Chloride intracellular channel 4(CLIC4)/Transforming Growth Factor Beta(TGF-β) signaling, while the vitamin K2 effect involved a hepatic Vitamin D Receptor(VDR)-phosphorylated AMPK signaling pathway. Fecal DNA sequencing by the Pacbio RSII method revealed there was significantly lower *Helicobacter apodemus*, *Helicobacter mesocricetorum*, *Allobaculum stercoricanis* and *Adlercreutzia equolifaciens* following both interventions compared to the model group. Moreover, different caecum acetic acid and butyric acid levels and enrichment of other specific microbes also determined the activity of the different regulatory pathways. Together these data show that *L. casei* Zhang and Vitamin K2 can suppress gut risk microbes and promote beneficial microbial metabolites to reduce colonic tumor development in mice.

## INTRODUCTION

Colorectal cancer (CRC) is a complex multifactorial digestive disease that is a major public health threat, accounting for the third most cancer-related mortalities, worldwide [1]. The human colon harbors one hundred trillion gut microbes and approximately 80% of the body's immune cells [2], and both are directly related to the pathogenesis of CRC. CRC have been revealed to be associated with microbiota dysbiosis [3], and this dysbiosis further accelerates CRC development [4]. Even susceptibility to CRC is associated with the composition of gut microbiota [5–6]. However, the relationship between regulation of host gene expression and the gut microbiome that is critical for CRC pathogenesis remain largely undefined, limiting mechanistic experimentation and the development of preventative and adjunct therapies.

As a microbiota modulator, probiotics from a healthy human gut can adhere to mucosa and colonize the colonic epithelium [7]. An abundance of data support the notion that probiotics might have protective effects for CRC [8]. Mechanistic findings have revealed that the use of probiotics could prevent CRC by reducing fecal enzyme activity [9], inducing cancer cell apoptosis and ameliorating inflammation [10–11], as well as increasing production of tumor-suppressive ferrichrome [12]. The action of probiotics may not be limited to these effects, as more systematic evaluations of probiotic are needed in CRC.

Over the past decade, the understanding of CRC-associated gut microbiota at the genus level was well-established with the development of culture-independent next generation sequencing (NGS) technology [13–14]. However, NGS was limited by short length reads with more operational taxonomic unit (OTU) inference, and the featured species could only be identified by sequencing the whole complex gut metagenome, which came at a high expense [15]. Recently, third generation sequencing technology based on whole length bacterial 16S rRNA sequencing has been shown to be a more precise tool for evaluating gut microbiota composition [16]. By using the Pacbio RSII system, CRC-associated gut microbiota were characterized at the species level.

In our previous work, we demonstrated that *Lactobacillus casei* Zhang could enrich *Bacteroides fragilis* and stimulate adiponectin receptor 2 signaling [17]. It has reported that vitamin K2 also enrich *Bacteroides* and vitamin K2-dependent osteocalcin can increase adiponectin expression[18–19]. Moreover, adiponectin exhibited anti-carcinogenic effects in colon tumorigenesis [20–21]. Thus, we hypothesized that *L. casei* Zhang and vitamin K2 might affect colon cancer by modulating the adiponectin. Furthermore, another study revealed that *Lactobacillus* fermented yoghurt can boost the absorption of menaquinone-7 in human, suggesting a synergistic effect of *Lactobacillus* and menaquinone-7 [22]. Considering all these, we tried to compare the mechanical similarities and differences of *L. casei* Zhang and Vitamin K2 by enrolling these two treatments. To determine whether specific microbes participate in the pathogenesis of CRC, we used third generation sequencing technology to quantify gut species. Additionally, we examined signaling molecules potentially involved in the anti-carcinogenic effect.

## RESULTS

**Figure 1:**
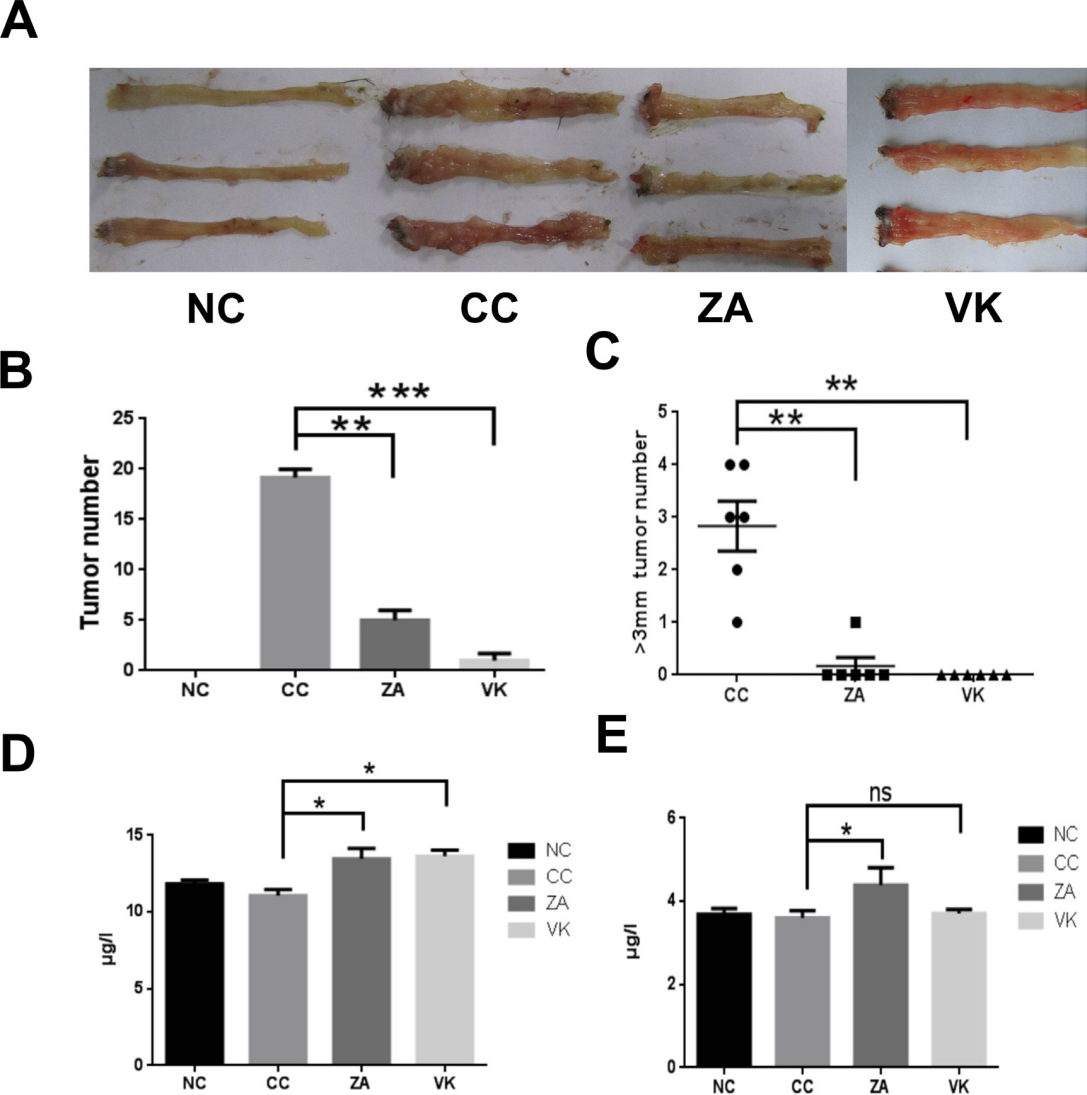
*L. casei* Zhang and Vitamin K2 prevent CRC development (**A**) Representative colonic polyps from the chemically-induced intestinal carcinogenesis model. (**B**) Comparison of colon polyp numbers from the NC, CC, ZA and VK mice (*n* = 6). (**C**) Comparison of the number of large colon tumors from the NC, CC, ZA and VK mice(*n* = 6). (**D**) Serum adiponectin levels from the different groups. (**E**) Serum osteocalcin levels from the different groups. Four groups: CC group was the colon cancer model, ZA group and VK group were treatment groups, and NC group as untreated healthy controls. Data are mean ± s.e.m *P < 0.05, **P < 0.01, ***P < 0.001, ns, No Significance.

To investigate whether *L. casei* Zhang or vitamin K2 protect mice against intestinal carcinogenesis, we subjected mice to a chemically-induced intestinal tumor protocol. In the AOM/DSS-induced model (CC), mice showed significantly increased tumor numbers and more tumors of large size compared with controls (Figure 1A and 1B). Expectedly, administration of *L. casei* Zhang or vitamin K2 decreased susceptibility to colon carcinogenesis, as fewer tumors and large-sized tumors were found (Figure 1B and 1C, p < 0.05). In addition, serum adiponectin levels were significantly increased after *L. casei* Zhang or vitamin K2 treatment compared with the model group (Figure 1D, *p* < 0.05). Furthermore, *L. casei* Zhang-treated mice also exhibited significantly increased osteocalcin levels compared with the CC group (*p* < 0.05), but the effect of vitamin K2 was modest (Figure 1E, *p* > 0.05).

**Figure 2:**
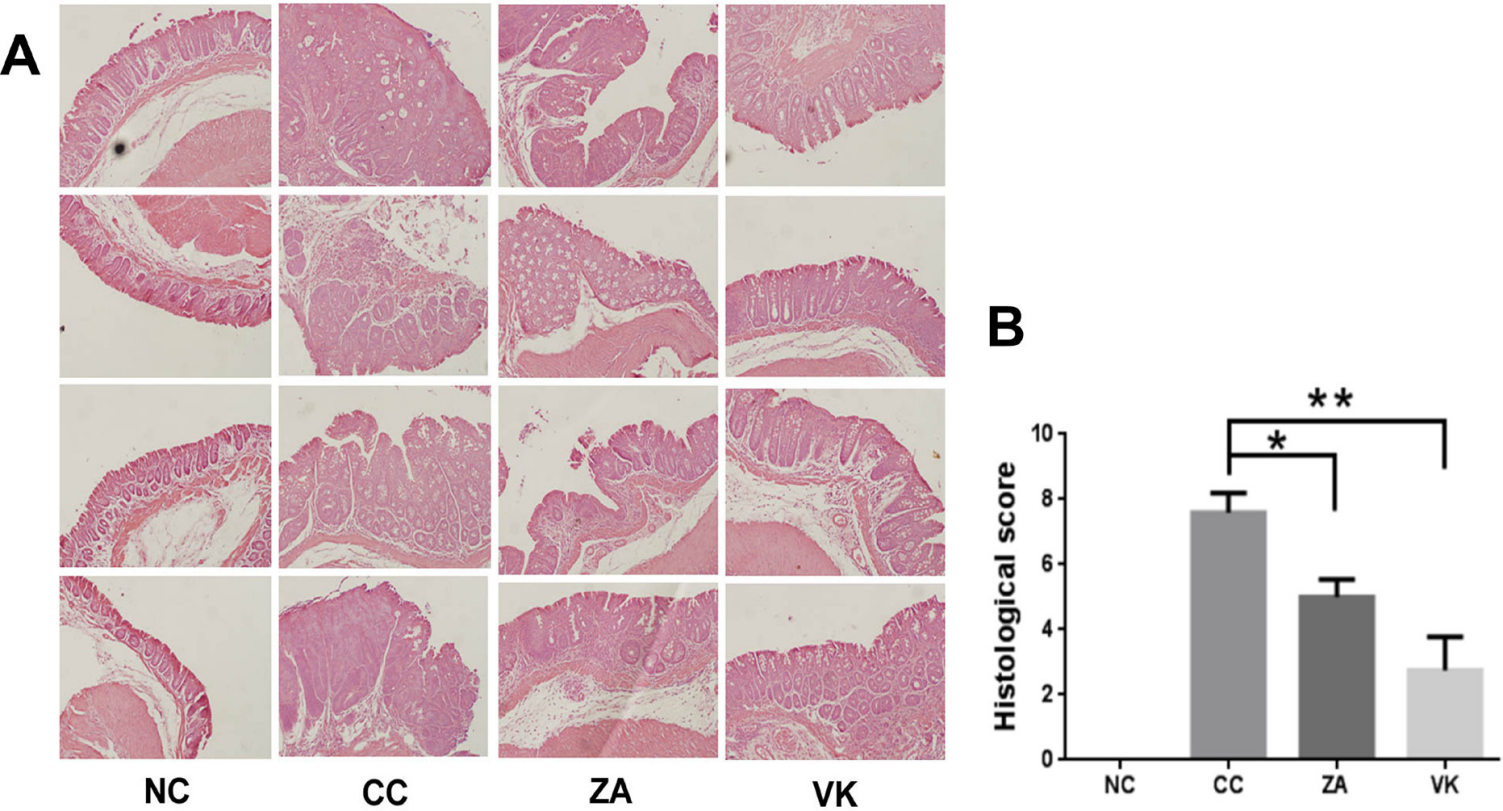
Representative histology slides and scores of the distal colon (**A**) HE staining of representative distal colon samples from NC, CC, ZA and VK mice. Magnification: 20×. (**B**) Histological scores of distal colon samples from NC, CC, ZA and VK mice. Data are mean ±s.e.m *P < 0.05, **P < 0.01.

In the evaluation of colon histology, the CC mice had severe inflammation and hyperplasia, while ZA and VK intervention resulted in reduced granulocytic infiltrate and hyperplasia, suggesting an attenuated response to the AOM/DSS treatment compared with the CC mice (Figure 2A). The histological score markedly decreased in treated mice (Figure 2B, *p* < 0.05), suggesting that administration of *L. casei* Zhang or vitamin K2 significantly reduced the severity of the AOM/DSS model.

**Figure 3:**
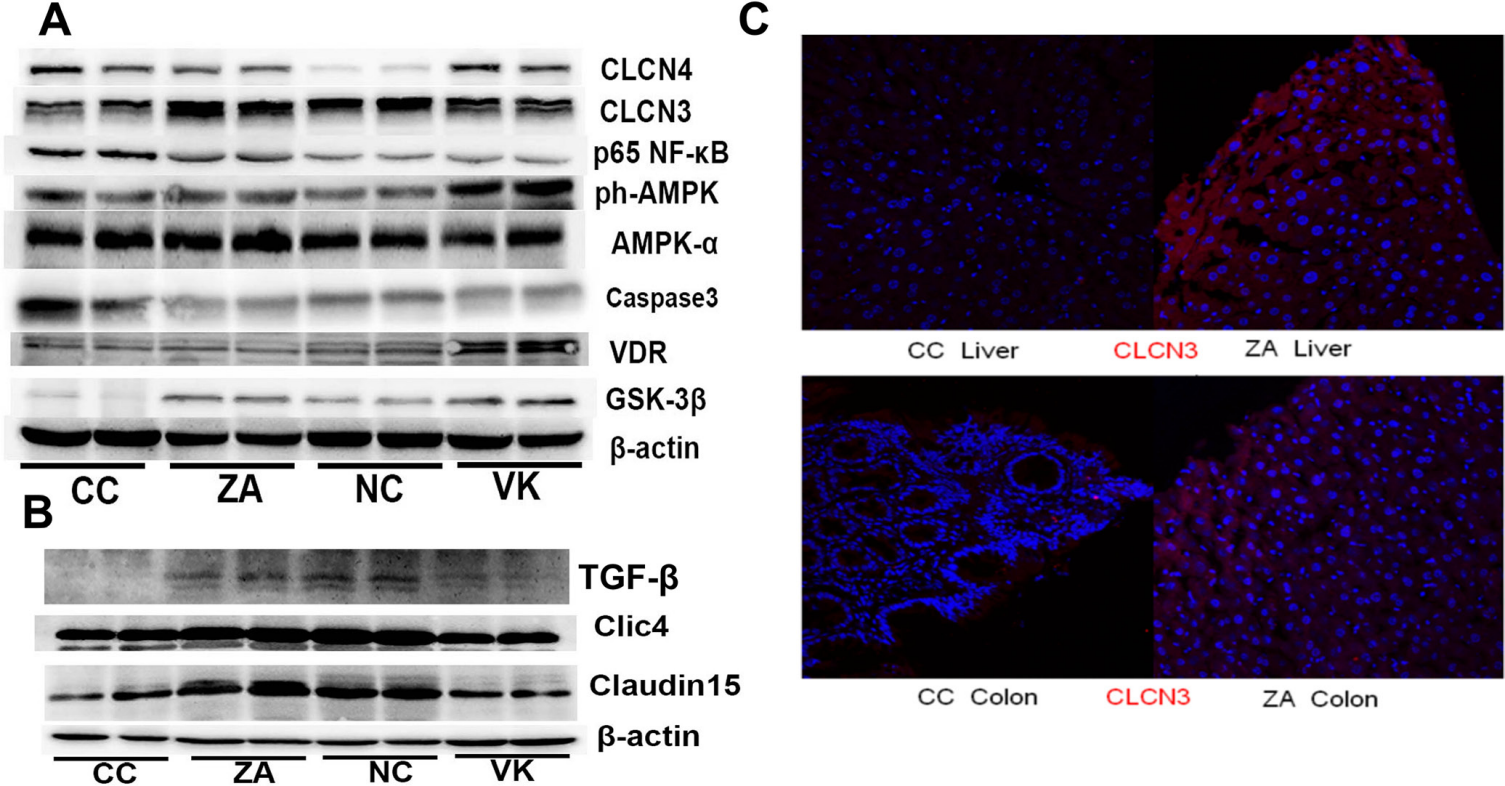
Liver and colonic expression of proteins involved in murine CRC (**A**) Immunoblot of hepatic CLCN3, CLCN4, NF-κB, AMPK, p-AMPK, VDR, GSK-3β and Caspase3 expression from NC, CC, ZA and VK mice. Equal amounts of protein extract 90 μg were loaded on each lane. (**B**) Immunoblot of colonic Claudin15, Clic4, TGF-β and β-actin from NC, CC, ZA and VK mice. (**C**) CLCN3 immunostaining in liver and colon tissues of representative CC and ZA mice. Immunostaining with anti-CLCN3 antibody (red), and 4′,6-diamidino-2-phenylindole (DAPI; blue) of fixed liver and colon sections.

*L. casei* Zhang or vitamin K2 administration markedly altered the expression of hepatic NF-κB, Caspase3 and GSK-3β, which are involved in chronic inflammation, cell proliferation and adiponectin regulation, respectively (Figure 3A). Expression of a variety of markers (CLCN4, p-AMPK, and VDR) was increased by vitamin K2 intervention, and CLCN3 was upregulated by *L. casei* Zhang (Figure 3A). The expression of Claudin15, Clic4, and TGF-β, were significantly higher in the colon tissue of ZA and NC mice compared with the colon tissue of CC mice (Figure 3B). Consistently, CLCN3 expression in liver and colon were enhanced in the ZA group compared with the CC group (Figure 3C).

**Figure 4:**
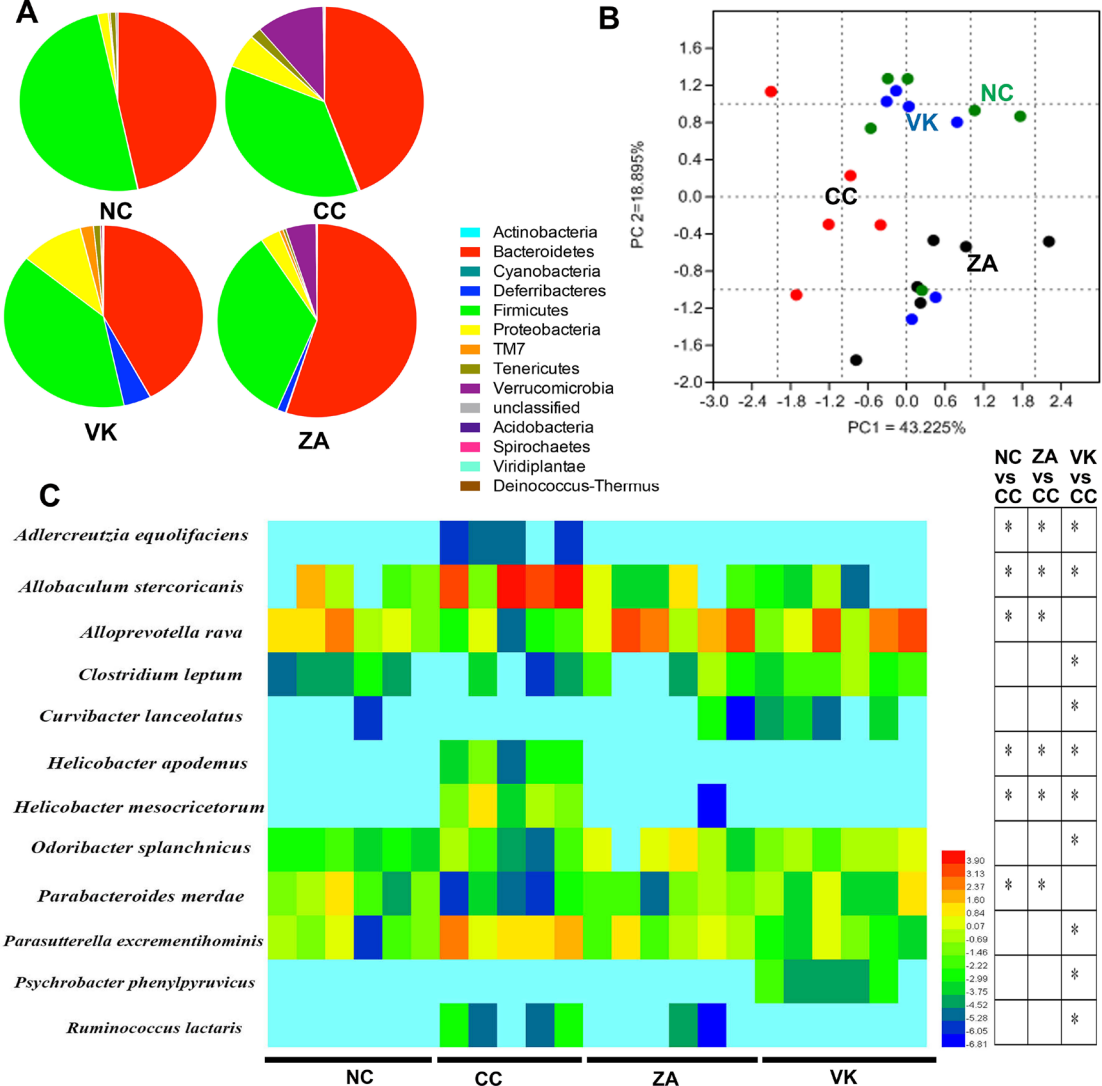
Altered bacterial microbiota biodiversity and composition in the different treatment groups (**A**) Comparison of the proportional abundance of gut microbiota at the phylum level. (**B**) Principal coordinate analysis of 16S rRNA sequences of gut microbiota within each sample (*n* = 5–6). PC1 and PC2 explain 43.225% and 18.895% of the variation, respectively. (**C**) Featured differences in the microbial community among the different treatment groups(*n* = 5–6). Data are mean ± s.e.m *P < 0.05.

Sequencing data for the whole 16S rRNA gene revealed variations in gut microbial composition following *L. casei* Zhang or vitamin K2 administration, suggesting that each intervention modulated the gut microbial community structure in distinctive ways (Figure 4A). Analysis at the phylum level revealed that the bacterial population of CC mice was characterized by an increased ratio of Bacteroidetes and Firmicutes and a high level of Verrucomicrobia and Proteobacteria (CC versus NC, Figure 4A). Vitamin K2 significantly increased the relative abundance of the Proteobacteria, increased the Deferribacteres, and decreased the Verrucomicrobia (VK versus CC, Figure 4A). Although *L. casei* Zhang also decreased the Verrucomicrobia and increased the Deferribacteres, probiotic-treated mice were characterized by an increase in the relative abundance of Bacteroidetes (ZA versus CC, Figure 4A). As shown in Figure 4B, principle component analysis showed a favorable separation of the CC and other sample groups. At the species level, colonic enrichment of *Adlercreutzia equolifaciens*, *Helicobacter apodemus* and *Helicobacter mesocricetorum* only in CC group combined with the high abundance of *Allobaculum stercoricanis* may predict increased risk for the development of sporadic CRC (Figure 4C). In addition, two enriched species (*Alloprevotella rava* and *Parabacteroides merdae*) were associated with *L. casei* Zhang administration, and there are six significantly enriched species after vitamin K2 administration, namely *Clostridium leptum*, *Curvibacter lanceolatus*, *Odoribacter splanchnicus*, *Parasutterella excrementihominis*, *Psychrobacter phenylpyruvicus* and *Ruminococcus lactaris*. Besides the enriched species, we further identified family Prevotellaceae was enriched in ZA group and *Lactobacillus* as a significantly enriched genera in the VK group (Supplementary Figure 1).

**Figure 5:**
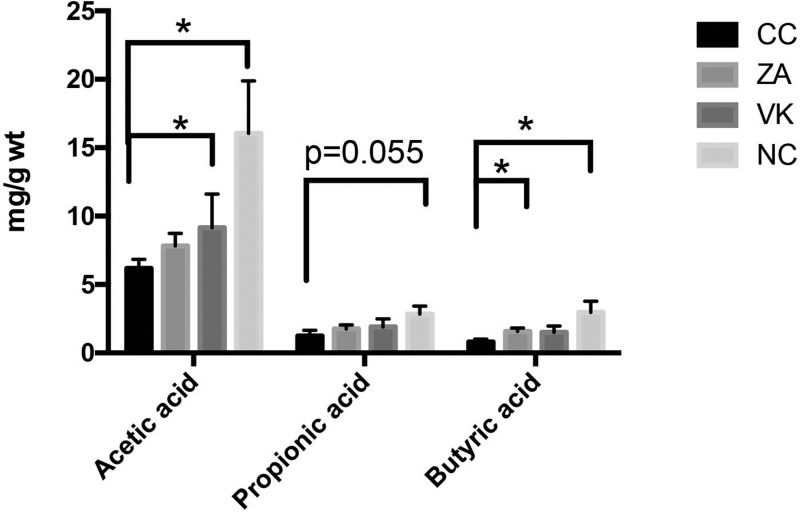
The effect of *L. casei* Zhang or Vitamin K2 on SCFAs, acetic acid, propionic acid and butyric acid production in caecum contents Data are mean ± s.e.m * *P* < 0.05.

We next investigated the effect of *L. casei* Zhang or Vitamin K2 on the production of SCFAs in caecum contents from the mice. Caecum acetic acid and butyric acid levels from the NC mice were significantly raised compared with the CC group (Figure 5, *p* < 0.05). *L. casei* Zhang and Vitamin K2 administration significantly enhanced the secretion of caecum butyric acid and acetic acid (Figure 5, *p* < 0.05).

## DISCUSSION

Human and animal studies have demonstrated that gut microbes and microbial products contribute to the etiology of CRC [23]. Here, we showed that both *L. casei* Zhang and MK-7 were able to preserve liver metabolic functions and delay polyp development in the AOM/DSS model of CRC. It is consistent that *Lactobacillus casei* ATCC334 and *Lactobacillus casei* Shirota also possess anti-tumorigenic effects via IL-6 inhibition and JNK-mediated apoptosis mechansims, respectively [12–24]. Adiponectin was one of the adipocytokines that reported to have anti-tumorigenic effects and was confirmed as a potential target for CRC therapy [25–26]. Our results demonstrated that both *L. casei* Zhang and MK-7 interventions are adiponectin-elevated, but might take actions via different signaling pathways. In parallel with the changes in adiponectin, GSK-3β expression was increased in both interventions. Interestingly, osteocalcin, a vitamin K2-dependent hormone, was promoted by *L. casei* Zhang but not MK-7. One explanation is that some but not all MK-n components may influence the production of osteocalcin.

There is not a single molecular-based mechanistic explanation for the actions of the probiotic *L. casei* Zhang in delaying CRC development. Potential polyps-promoting microbes including *Helicobacter apodemus*, *Helicobacter mesocricetorum*, *Allobaculum stercoricanis* and *Adlercreutzia equolifaciens* flourished in the CRC group and *L. casei* Zhang reduced the risk from these microbes. At the phyla level, *L. casei* Zhang induced an increased Bacteroidetes/Firmicutes ratio, and a decreased level of Verrucomicrobia. To our knowledge, the role of *Helicobacter apodemos* and *Helicobacter mesocricetorum* in risk of CRC has not been addressed. Expansions of *Allobaculum* have demonstrated in patients with anastomotic injury and *Allobaculum stercoricanis* may also be involved in the intestinal injury of CRC [27]. Recent investigations suggest that *Adlercreutzia equolifaciens* is an equol-producing bacteria, and equol could bind to estrogen receptors with anti-tumorigenic effects [28]. In contrast, *A. equolifaciens* was positively correlated with cancer risk, and it has been hypothesized that *A. equolifaciens* might be a probiotic passenger bacteria with a competitive advantage in the tumor niche for the gradual replacement of driver bacteria in the later oncogenic process according to the colorectal carcinogenesis “driver-passenger” model [29].

SCFAs, especially butyric acid, could induce activation of regulatory T cells (Tregs) via TGF-β signaling [30]. In this study, colonic TGF-β/CLIC4 signaling, negatively associated with CRC oncogenesis [31], stimulated by high butyric acid levels in NC and *L. casei* Zhang-treated mice. Furthermore, CLIC4 has been proven to suppress tumor cell growth by enhancing TGF-β responsiveness [32]. In addition, greater relative abundance of *Prevotella* from Prevotellaceae family seen in the population of primitive tribes was consistent with production of SCFAs [32], while ZA group matched an enrichment of *Alloprevotella* from Prevotellaceae family with higher butyric acid level suggesting a potential SCFAs-producing role of *Alloprevotella*. On the other hand, the chloride ion also triggered another anti-inflammatory gene, CLCN3 [33]. Another study showed that *Parabacteroides* enrichment could maintain IL-10-producing Treg to reduce intestinal inflammation [34]. In summary, anti-inflammatory CLCN3 signaling and high levels of *Parabacteroides merdae* combined with anti-oncogenic TGF-β/CLIC4 signaling exhibited an anti-tumor effect. Moreover, we also found that caecum butyric acid levels and active TGF-β signaling in the gut led to cancer prevention. But the precise butyric acid producing species was not traced. Finally, Claudin15, which is relevant to *Bacteroides* [35], is associated with CLIC4 expression and might contribute to intestinal integrity.

AMPK signaling is highly complex and one of the most central signaling pathways in the cell. A synergistic preventive effects against colon cancer was observed with Vitamin D3 and the AMPK agonist metformin [36]. And VDR signaling has been associated with gut microbiota and chronic inflammation [37–38]. However, what is upstream of VDR signaling and its relation to gut microbes has not been fully explored. In our study, we for the first time found that the microbial product MK-7 was effective in stimulating VDR expression, suggesting that gut microbiota might partially influence host VDR signaling via its metabolite vitamin K2. Furthermore, risk microbes such as *H. Apodemus* and *H. mesocricetorum* were also reduced by vitamin K2 administration. In addition, *Lactobacillus* spp. was more abundant in the MK-7 group than the CC group, which demonstrated anti-tumorigenic effects. Guts of MK7-fed mice, which developed fewer colonic tumors compared with CC mice, were highly enriched in *Proteobacteria*, including increased relative abundance of *Parasutterella excrementihominis*, *Curvibacter lanceolatus* and *Psychrobacter phenylpyruvicus*. Consistently, a previous study found that *Parasutterella* decreased in CRC patients compared with healthy volunteers [13]. However, to date, these bacteria remain poorly explored.

In conclusion, this work demonstrated the preventive effects of *L. casei* Zhang and MK-7 in CRC. The anti-tumorigenic effects not only shared mechanisms, inhibiting CRC-risk microbes and enhancing adiponectin secretion, but also increased some specific gut microbes and triggered different anti-inflammatory and anti-oncogenic pathways. Our data showed gut microbiota and its product vitamin K2 determined the susceptibility to colon cancer via a complex mechanism. Additional studies are required to further characterize the bacteria or metabolites that induce regulatory signals in CRC tumorigenesis.

## MATERIALS AND METHODS

### Animals and treatments

Male C57BL/6J mice were purchased and maintained in the Animal Experimental Center of Third Military Medical University (Chongqing, China) under specific pathogen-free conditions with controlled temperature (22°C ± 2°C) and humidity (55% ± 5%) as well as a 12/12 h light/dark cycle. All mice were allowed free access to standard sterile mouse chow and water. Investigation has been conducted in accordance with the ethical standards and according to the National Institutes of Health Guide for the Care and Use of Laboratory Animals. All protocols were approved by the Animal Care and Use Committee at Third Military Medical University. After acclimation for 7 d, mice were divided into four groups, i.e., the colon cancer group (6 mice,CC, Figure 1), *L. casei* Zhang with cancer induction group (6 mice,ZA, 4 × 10^9^ cfu/d), Vitamin K2 with cancer induction group (6 mice,VK, MK-7 addition was 50 mg/kg diet), and untreated controls (6 mice, NC group).

### Induction of colitis-associated colon cancer

Chronic colitis-associated colon cancer was induced using the well-established Azoxymethane/Dextran sulfate sodium (AOM/DSS) model [39]. Briefly, mice received a single intraparietal injection of AOM ( 12.5 mg/kg body weight, Sigma,). After 1 week, the AOM-injected mice were given 2.5% DSS (36–50 kDa; MP Biomedicals) in the drinking water for 5 d, followed by plain water for 16 d. The DSS/plain water treatment was repeated for three cycles. Weight change was monitored and tumor numbers were counted after sacrifice.

### Concentrations of serum adiponectin and osteocalcin

Serum was centrifuged at 3500 × g for 15 min at 4°C and frozen at −80°C for further analysis. ELISA for adiponectin and osteocalcin levels was conducted on murine serum according to the manufacturer's instructions (Millipore Inc.).

### Measuring caecum short-chain fatty acids (SCFAs)

SCFAs were measured by gas chromatography from caecum contents as described previously [40]. A 1:5 dilution of caecum in 1 M HCl was centrifuged (12000 ×*g*, 15 min), and diethylacetic acid (Sigma) was added to a final concentration of 1 mM as an internal standard. The supernatant was *filtered* through a 0.22-μm membrane and injected for measurement on an Agilent 6890N GC system equipped with a flame ionization detector and an automatic liquid sampler (Agilent Technologies, Santa Clara, CA, USA).

### Sequencing the 16S rRNA gene

Total bacterial DNA was extracted and purified using the QIAGEN mini stool kit (QIAGEN, Valencia, CA, USA) and AMP beads (Pacific Biosciences). Subsequent amplification of the bacterial 16S rRNA region was performed using SMRT barcode sequencing PCR primers. DNA libraries were constructed by the Pacific Biosciences SMRT bell template prep kit 1.0. The pooled 16S rRNA PCR amplicons were sequenced on a Pacbio RSII system as previous described [41]. OTU was classified using Quantitative Insights into Microbial Ecology (QIIME) package (version 1.7). The Wilcoxon–Mann–Whitney test was performed to compare data at various OTU levels. The weighted and unweighted principal coordinate analysis (PCoA) was undertaken based on the UniFrac distances.

### Western blot analysis

T-PER Kit (Thermo Scientific) was used to extract total protein from fresh-frozen liver and colon tumor tissues (50 mg per sample). Protein lysates were loaded equal amount of 90μg on each lane and separated by SDS-PAGE and transferred to PVDF membranes. After blocking in 10% skim milk for 1 h, blots were probed with antibodies directed against CLCN3 (Abcam), CLCN4 (Sigma), NF-κB (Abcam), AMPK (Cell Signaling Technology), p-AMPK (Cell Signaling Technology), VDR (Proteintech), GSK-3β (Abcam), Caspase3 (Abcam), Claudin15 (Santa Cruz), Clic4 (Abcam), TGF-β (Cell Signaling Technology) and β-actin (Cell Signaling Technology). Protein bands were visualized by a chemiluminescence system (Tanon, Beijing, China) with Millipore Immobilon ECL.

### Histological and immunofluorescent staining

The distal colon (0.5 cm) was cut open longitudinally and fixed in 4% paraformaldehyde for 48 h. After gradient dehydration, samples were cut into 5 μm-thick serial sections for hematoxylin and eosin (HE) staining. All HE slides were observed on an optical microscope (Olympus, Tokyo, Japan). Histological score was evaluated blindly according to a previous research with minor modification [42]. Brifely, inflammation Severity(30%), ulceration(30%) and hyperplasia(40%). And each field was devided into 5 grade levels(0, 1, 2, 3, 4).

For immunofluorescent staining, tissues were fixed in PBS containing 4% paraformaldehyde for 48 h, followed by gradient dehydration with a sucrose solution overnight and frozen embedding with OCT. Embedded tissues were cut into 10 μm-thick sections, and slides were incubated with primary antibody at 4°C overnight, then stained with Alexa Fluor 568 goat anti-rabbit IgG (Invitrogen) for CLCN3 detection at room temperature for 2 h. Slides were further stained with DAPI (CST) for Nuclei. All slides were observed on a confocal microscope (Olympus).

### Statistical analysis

Experiments were performed with more than five mice per group. Data are presented as means ± SEM. One-way ANOVA followed by LSD test was used for statistical significance determination using the Prism 7 software (Graphpad, La Jolla, CA, USA). Significance was set at *p* < 0.05.

## SUPPLEMENTARY MATERIALS FIGURE


