# Supplementary material for: Lactobacillus casei Zhang and vitamin K2 prevent intestinal tumorigenesis in mice via adiponectin-elevated different signaling pathways
Source: Oncotarget. 2017 Mar 1;8(15):24719–27. doi: 10.18632/oncotarget.15791 (PMC5421882; doi:10.18632/oncotarget.15791)
Supplement: Supplementary file 1 [file oncotarget-08-24719-s001.pdf]

Lactobacillus casei Zhang and vitamin K2 prevent intestinal tumorigenesis in mice via adiponectin-elevated different signaling pathways

Supplementary Materials

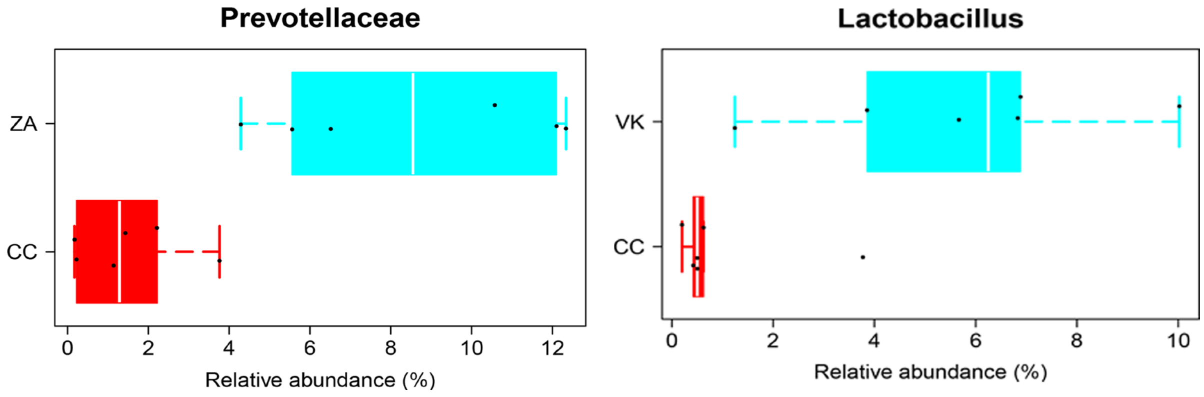

Supplementary Figure 1: Prevotellaceae family enriched in the ZA group and *Lactobacillus* genera enriched in the VK group.
